# Determinants of hypertension-related knowledge, attitude, and practices (KAP) among caregivers in Neno, rural Malawi: A cross-sectional study

**DOI:** 10.1016/j.heliyon.2024.e41546

**Published:** 2024-12-27

**Authors:** Chikondi Maluwa, Sitalire Kapira, Hataichanok Chuljerm, Wason Parklak, Kanokwan Kulprachakarn

**Affiliations:** a School of Health Sciences Research, Research Institute for Health Sciences, Chiang Mai University, Chiang Mai, Thailand; b Ministry of Health, Neno District Health Office, Malawi; c Partners in Health, Neno Office, Malawi; d Research Center for Non-infectious Diseases and Environmental Health, Research Institute for Health Sciences, Chiang Mai University, Chiang Mai, Thailand

**Keywords:** Hypertension, Knowledge, attitude, and practices (KAP), Caregivers, Malawi

## Abstract

**Background:**

Hypertension, a significant health concern, increases the risk of cardiovascular disease and premature mortality. Caregivers play a crucial role in ensuring optimal care for hypertensive patients and reducing associated complications. Caregivers' basic knowledge, good attitude, and relevant practices are necessary to ensure high-quality care for patients with hypertension. However, there is no research conducted in Malawi that investigated the knowledge, attitude, and practices of caregivers towards hypertension prevention and management.

**Objective:**

The study aimed to assess knowledge, attitude, and practices towards hypertension and their determinants among caregivers of hypertensive patients in Neno, Malawi.

**Methods:**

Our study, conducted in Neno, Malawi, involved 422 caregivers of hypertensive patients. We used a cross-sectional study design. Data was collected through a structured questionnaire and analyzed using SPSS Version 22.0.

**Results:**

The participants had a mean age of 44.94 years (SD = 9.889), with 63.3 % being female. The mean KAP scores were 38 %, 93.3 %, and 78.7 %, respectively. Positive correlations were found between knowledge and practice (r = +0.252; p < 0.001) and knowledge and attitude (r = +0.255; p < 0.001). However, no significant relationship was observed between attitude and practice (r = +0.064; p = 0.190). Age showed a strong correlation with attitude (r = +0.233; p < 0.001) but not with knowledge or practice. On the other hand, occupation, education level, and caregiver-patient relationship significantly influenced knowledge and attitude but not practice. Gender also demonstrated a notable association with KAP regarding hypertension.

**Conclusion:**

Caregivers demonstrated poor knowledge but engaged in good practices. Despite their limited understanding, they maintained an excellent attitude towards hypertension. This highlights the necessity for increased prevention, and control strategies within communities, emphasizing health education on lifestyle modifications and to address the gaps identified in caregivers’ understanding in the prevention and management of hypertension.

## Highlights

• Caregivers of hypertensive patients have limited knowledge towards hypertension with good attitude and poor practices.
• Caregivers' attitude towards hypertension does not have any impact on their practices.
• Duration in care of the patient has no influence on the knowledge, attitude, and practices of the caregivers.

## 1 Introduction

Hypertension is a chronic, non-communicable condition that affects 1.13 billion people worldwide and is a major public health concern [1]. Though it is a non-communicable disease on its own, it is also one of the risk factors for developing cardiovascular diseases. Given the cost of medical treatment in low-resource countries, prevention efforts should prioritize reducing reliance on expensive treatments for NCDs. Healthcare systems need to take a more public health-oriented approach to persuade people to make healthier lifestyle choices and prevent the development of NCDs [2].

In Malawi, hypertension is particularly a great concern, with a prevalence of 33 %, the highest in the sub-Saharan region. Key risk factors for hypertension include advanced age, tobacco and heavy alcohol use, obesity, physical inactivity, and poor dietary habits characterized by high salt and sugar intake coupled with low consumption of fruits and vegetables [3]. According to a World Health Organization (WHO) report titled "Global report on hypertension: The race against a silent killer," hypertension is one of the most serious killers of modern times, killing 108 000 Malawians annually or roughly 296 every day [4].

Caregivers, primarily family members in Africa, play a crucial role in managing hypertensive patients. Unlike in developed countries where professional healthcare providers may dominate patient care, family caregivers in LMICs are typically the primary support system for individuals living with chronic conditions [5]. Caregivers are essential in managing hypertension by reminding patients to take medications, assisting with household tasks, providing social and physical support, and monitoring blood pressure. To improve patient outcomes, caregivers need better support and access to healthcare resources, as their knowledge, attitudes, and practices (KAP) significantly affect patient outcomes [6]. Global studies show varying KAP levels among caregivers based on regional healthcare systems. Research from Jordan showed good caregivers knowledge about cardiovascular diseases [7] which is similar to research from Bangladesh which indicated fair general knowledge with negative attitude but fair general practices among caregivers [8]. This is contrary to research from India and Ghana which highlighted gaps in caregivers' knowledge about hypertension [9,10]. Similarly, studies from Malaysia, China, Brazil and Zimbabwe indicate that communities have limited knowledge about hypertension and other NCDs [[11], [12], [13], [14]].

In Malawi like most of the LMICs, caregivers play a vital role in providing ongoing care for hypertensive patients in the absence of a health care provider. The standard of care they provide depends on their knowledge, influencing their attitude and practices toward preventing and managing hypertension [15]. Assessing caregivers' knowledge, attitude, and practices is therefore important to pinpoint areas where focused interventions may be required. By focusing on this group, we intend to highlight the important role that informal caregivers play in health outcomes within low- and middle-income countries (LMICs).

Thus, the study aimed to assess knowledge, attitude, and practices towards hypertension among caregivers of hypertensive patients and to identify factors that influence the KAP. As there are few research that were conducted in sub-Saharan Africa on hypertensive patients' caregivers’ KAP, this is essential for developing public health strategies that promote caregiver education and support, ultimately reducing the burden of hypertension and other non-communicable diseases (NCDs) while improving health outcomes for affected individuals in resource limited areas like Malawi.

## 2 Materials and methods

### 2.1 Study design, area, and population

The study used a cross-sectional study design in rural setting in Neno, Malawi. Our study included caregivers who were aged 18 years or older and who have been caring for hypertensive patients for at least six months. This inclusion criterion was chosen to ensure that the caregivers had sufficient experience to provide meaningful insights into their knowledge, attitudes, and practices related to hypertension prevention and management. Caregivers who had a history of hypertension or any other chronic illness; who had cognitive impairments that may affect their ability to comprehend the study questions; who had a history of psychiatric illness; and those who did not provide their consent were excluded.

### 2.2 Sampling technique

Systematic random sampling technique was employed in this study. The participants were selected from the Integrated Chronic Care Clinic (IC3) at Neno District Hospital and Lisungwi Community Hospital. These caregivers accompanied their patients to the clinic for scheduled appointments. Upon arrival, both caregivers and patients attended health education sessions that were not related to hypertension. After these sessions, they waited in line to consult with healthcare service providers. Once their appointments were concluded, we invited every third caregiver for an interview.

### 2.3 Sample size

The sample size was calculated using the formula: n = [Z^2 ∗ P ∗ (1-P)] / E^2

Where: n = sample size, Z = the z-score for the level of confidence (1.96 for 95 %), P = expected proportion or prevalence, E = the margin of error (0.05) [16]. Assuming a prevalence of 50 % for adequate knowledge, attitude, and practices, and a margin of error of 5 %, the sample size was 384. Considering a 10 % non-response rate, there were 422 respondents selected in total for this study [17].

### 2.4 Research tool and data collection

The period of data collection was from November 27, 2023 to December 22, 2023. A standardized questionnaire was used to conduct an interview. A literature research and expert views were used to design a set of KAP questionnaires [14,[18], [19], [20], [21], [22], [23], [24], [25], [26]]. Before being used, the questionnaire was evaluated and approved by the Research Institute for Health Sciences questionnaire approval committee. The questionnaire was produced in English and translated into Chichewa, the native language for Malawi for easy communication. The questionnaire was divided into two sections: one on sociodemographic characteristics about the respondents and the other on knowledge, attitude, and practice towards hypertension. The respondents' location, religion, relationship with the patient, age, education level, gender, and date of diagnosis (duration in care) were all included in the first section of the sociodemographic information. This was collected to compare KAP of hypertension and determine the factors influencing KAP of hypertension within the study population. Nine questions covering signs and symptoms, prevention, complications, risk factors, and sources of information about hypertension were included in the second section of the knowledge test. The options for responses were "yes" and "no." Likert-scale questions were utilized in this section on attitudes and practices. There were six questions on attitudes, with three scales: "agree," "not sure," and "disagree." There were eight practice questions with the options to respond "always," "sometimes," "rarely," or "never." Prior to the study, the questionnaire's appropriateness, validity, and reliability were assessed in a pilot study, and any necessary improvements were made. To facilitate communication between the participants and the researcher, the interview was done in the native Chichewa language of the respondents.

### 2.5 Data management and statistical analysis

In the knowledge section, there were 7 questions. Every correct answer was granted 1 point and each wrong answer a 0. Those who managed to get half of the answers and above correctly on each question were considered to have correctly answered the question while those who got one answer right and above but less than half were considered to have partially correctly answered the question and those who got a zero were considered to have wrongly answered the question and the total score was 25. In the attitude section, the total score was 18 from 6 questions. A 3-point Likert scale was adopted: 1 for “Disagree”, 2 for “Not sure”, and 3 for “Agree”. The same as practice section, a 4-point Likert scale was adopted:1 for “Never”, 2 for “Rarely”, 3 for “Sometimes”, and 4 for “Always” with a total score of 32 from 8 questions.

The data was imported from Microsoft Excel to Statistical Package for Social Science (SPSS) version 22.0. To determine the total KAP scores for each respondent about hypertension, the answers to each question were scored appropriately and totaled for each section. Descriptive statistics including counts, percentages, and proportion for all categorical variables were calculated. The overall score was utilized to calculate the KAP level in relation to hypertension and its contributing factors. The correlation between KAP was analyzed using Pearson correlation test while the association of KAP with age and duration in care was examined using Bivariate analysis, and the association of the KAP with various groups of sociodemographic variables was examined using Mann-Whitney *U* test. Results were considered significant at *p* < 0.05.

### 2.6 Ethical approval

The study was approved by the Research Institute for Health Sciences (RIHES), Chiang Mai University Ethics Committee, Thailand [Project No.16/66, Date: 5/10/2023], and Malawi National Health Sciences Research Committee [Protocol # 23/10/4216, Date: 23/11/2023]. We also got permission from the Neno District Health Office to conduct the study in Neno. The respondents were informed about the study and provided their written informed consent prior to the interview. The respondents were free to discontinue their involvement in the study at any time, as it was entirely voluntary. Additionally, they were free to decline to respond to any questions that made them uncomfortable. All their privacy was protected during the research.

## 3 Results

### 3.1 Sociodemographic characteristics of hypertensive patients’ caregivers

Among the 422 interviewed caregivers, 267 (63.3 %) were females, 54 (12.8 %) were from 18 to 34 years, and 76 (18 %) were above 54 years. The mean age of participants was 44.9 years (SD = 9.9). More than half (63.0 %, n = 266) of the caregivers were spouses, and 25.8 % (n = 109) were children. Most of the caregivers, 63.7 % (n = 269) had at least primary education. Most caregivers were farmers (58.5 %, n = 247). All the participants were Christians, with more than half (61.4 %, n = 259) of their patients having been in care for 1–5 years. Additionally, most of them (97.8 %, n = 413) got their knowledge from a healthcare provider (Table 1).

**Table 1:** Socio-demographic characteristics of hypertensive patients’ caregivers (n = 422).

**Characteristics**	**Males n (%)**	**Females n (%)**	**Total n (%)**
**Location**			
Neno Hospital	65 (15.4 %)	109 (25.8)	174 (41.2)
Lisungwi	90 (21.4)	158 (37.4)	248 (58.8)
**Age**			
18–34	15 (3.6 %)	39 (9.2 %)	54 (12.8 %)
35–44	40 (9.5 %)	125 (29.6 %)	165 (39.1 %)
45–54	62 (14.7 %)	65 (15.4 %)	127 (30.1 %)
>54	38 (9 %)	38 (9 %)	76 (18 %)
**Level of education**			
No education	4 (0.95)	14 (3.32 %)	18 (4.3 %)
Primary	83 (19.6 %)	186 (44.1 %)	269 (63.7 %)
Secondary	64 (15.2)	58 (13.7 %)	122 (28.9)
Tertiary	4 (0.95 %)	9 (2.13 %)	13 (3.1 %)
**Caregiver-patient relationship**			
Child	20 (4.73 %)	89 (21.1 %)	109 (25.83 %)
Spouse	117 (27.72 %)	149 (35.31 %)	266 (63.03 %)
Parent	17 (4.03 %)	27 (6.4 %)	44 (10.43 %)
Others	1 (0.24 %)	2 (0.47)	3 (0.71 %)
**Location**			
Neno Hospital	65 (15.4 %)	109 (25.8)	174 (41.2)
Lisungwi	90 (21.4)	158 (37.4)	248 (58.8)
**Patient's date of Diagnosis/Duration in care**			
<1 year (6–11 months)	23 (5.5 %)	24 (5.7 %)	47 (11.2 %)
1–5 years	88 (20.9 %)	171 (40.5)	259 (61.4)
6–10 years	33 (7.8 %)	66 (15.6 %)	99 (23.4 %)
>10 years	11 (2.6 %)	6 (1.4 %)	17 (4 %)
**Occupation**			
Farmers	72 (17.1 %)	175 (41.4 %)	247 (58.5 %)
Business	65 (15.4 %)	58 (13.7 %)	123 (29.1 %)
Employed	14 (3.3 %)	21 (5 %)	35 (8.3 %)
None	4 (0.95 %)	13 (3.1 %)	17 (4 %)
**Source of knowledge**			
Health care provider	150 (35.5 %)	263 (62.3 %)	413 (97.8 %)
Internet	9 (2.1 %)	8 (1.9)	17 (4 %)
Support group	0 (0 %)	0 (0 %)	0 (0 %)
CHW	3 (0.71 %)	12 (2.84 %)	15 (3.55 %)
Others	2 (0.47)	1 (0.24)	3 (0.71)

### 3.2 Knowledge regarding hypertension

The participants had poor knowledge score of 38 %, median of 9.00, a total mean and standard deviation of 9.5 (SD = 1.9) and minimum and highest scores of 5 and 19, respectively out of a total score of 25. Out of all the participants, 10.2 % (n = 43) correctly answered the risk factors of hypertension, while the rest responded partially correctly, with 100 % of the participants mentioning excessive salt intake as one of the risk factors while 33.4 % (141) mentioned excessive alcohol intake as one of the risk factors. On complications, 3.1 % (n = 13) correctly answered, and the rest partially responded correctly, and all the participants mentioned stroke as one of the complications. 15.4 % (n = 65) answered correctly on the prevention of hypertension, and the rest of the respondents partially answered correctly. Minimal salt intake was mentioned by all the participants as one of the prevention measures. Less than half of the respondents, 46.2 % (n = 195), knew the normal measurement of the blood pressure reading. However, 1.0 % (n = 4) participants were able to know how often the blood pressure should be monitored, with 96 % (n = 405) partially having an idea on how often the blood pressure should be monitored (Table 2).

**Table 2:** Knowledge score about hypertensive among caregivers along with the respective mean, median and standard deviation (n = 422).

**Characteristics**	**Correct, n (%)**	**Partially correct, n (%)**	**Wrong n (%)**	**Mean**	**Median**	**SD**
**What is hypertension**						
Males	10 (2.4)	141 (33.4)	4 (1.0)	1.0	1.0	0.2
Females	7 (1.7)	259 (61.4)	1 (0.2)			
**Risk Factors of Hypertension**						
Males	18 (4.3)	137 (32.5)	0 (0.0)	2.5	2.0	0.9
Females	25 (5.9)	242 (57.3)	0 (0.0)			
**Signs and symptoms of hypertension**						
Males	95 (22.5)	59 (14.0)	1 (0.2)	1.5	2.0	0.5
Females	124 (29.4)	139 (32.9)	4 (1.0)			
**Complications of hypertension**						
Males	4 (1.0)	151 (35.8)	0 (0.0)	1.2	2.0	0.5
Females	9 (2.1)	258 (61.1)	0 (0.0)			
**Prevention of hypertension and its complications**						
Males	20 (4.7)	135 (32.0)	0 (0.0)	1.9	1.0	0.7
Females	45 (10.7)	222 (52.6)	0 (0.0)			
**How often should blood pressure be monitored**						
Males	1 (0.2)	154 (36.5)	0 (0.0)	1.0	1.0	0.2
Females	3 (0.7)	251 (59.5)	13 (3.1)			
**What is a normal BP reading**						
Males	92 (21.8)	0 (0.0)	63 (14.9)	0.5	0.0	0.5
Females	103 (24.4)	0 (0.0)	164 (38.9)			

Note: SD: Standard Deviation.

### 3.3 Attitude regarding hypertension

Minimum and maximum attitude scores were 13 and 18, respectively, and excellent attitude score of 93.3 %, with a median of 17 and interquartile range (IQR) of 2 (16–18). Of those who took part, 415 (93.8 %) believed that hypertension was a serious condition. Nevertheless, 95 % (n = 401) of the participants thought that hypertension can be managed with medication and lifestyle changes, and 96.6 % (n = 408) agreed that caregivers play an important role in the management of hypertension. Among the participants, 55.2 % (n = 233) felt confident in their ability to take care of a hypertensive patient, while 39.1 % (n = 165) were not sure, and 5.7 % (n = 24) disagreed with having confidence in caring for the hypertensive patient. 96.9 % (n = 409) felt supported by the health care professional in caring for the patient. However, 53.6 % (n = 226) participants thought that they had enough information about hypertension while 34.8 % (n = 147) were not sure if they had enough information and 11.6 % (n = 49) disagreed having enough information about hypertension (Table 3).

**Table 3:** Distribution of caregivers’ responses to attitude items about hypertension along with median and interquartile range (IQR) (n = 422).

**Attitude Items**	**Frequency**	**Percent**	**Median**	**IQR**
**Included in Scoring System for Attitude**				
**Hypertension is a serious condition**				
Agree	415	98.3	3	0 (3–3)
Disagree	2	0.5		
Not sure	5	1.2		
**Hypertension can be managed with medication and lifestyle changes**				
Agree	401	95.0	3	0 (3–3)
Disagree	2	0.5		
Not sure	19	4.5		
**Caregivers play an important role in managing hypertension**				
Agree	408	96.7	3	0 (3–3)
Disagree	2	0.5		
Not sure	12	2.8		
**You are confident in your ability to manage the patient's hypertension**				
Agree	233	55.2	3	1 (2–3)
Disagree	24	5.7		
Not sure	165	39.1		
**You are supported by healthcare professionals in managing the patient's hypertension**				
Agree	409	96.9	3	0 (3–3)
Disagree	2	0.5		
Not sure	11	2.6		
**You have enough information about hypertension to effectively care for a hypertensive patient**				
Agree	226	53.6	3	1 (2–3)
Disagree	49	11.6		
Not sure	147	34.8		

Note: IQR: Inter quartile range.

### 3.4 Practice regarding hypertension

The minimum and maximum practice scores were 14 and 32, respectively, and the participants exhibited good practice with an overall mean score of 78.7 %, median of 25 and interquartile range of 3 (24–27). Among the participants, 74.6 % (n = 315) always made sure that patients took their medication as prescribed by the doctor, while 33.9 % (n = 143) of the participants always encouraged patients to make lifestyle changes. Nonetheless, 17.5 % (n = 74), 69.9 % (n = 295), 60.2 % (n = 254) and18.7 % (n = 79) of participants consistently monitored blood pressure regularly, always kept the blood pressure readings, always communicated with the health care professionals about the patient's management and always accompanied the patient to the doctor's appointment, respectively. Almost half (50.7 %, n = 214) of the participants rarely helped the patient with meal planning, and 44.3 % (n = 187) sometimes prepared meals for the patient that met hypertension's dietary guidelines (Table 4).

**Table 4:** Distribution of caregivers’ responses to practice items about hypertension along with median and interquartile range (IQR) (n = 422).

**Practice Items**	**Frequency**	**Percent**	**Median**	**IQR**
**Included in Scoring System for Attitude**				
**Do you ensure that the patient takes their medication as prescribed?**				
Always	315	74.6	4	1 (3–4)
Never	0	0		
Rarely	12	2.8		
Sometimes	95	22.5		
**Do you encourage the patient to make lifestyle changes such as exercising and eating a healthy diet?**				
Always	143	33.9	3	1 (3–4)
Never	2	0.5		
Rarely	15	3.6		
Sometimes	262	62.1		
**Do you monitor the patient's blood pressure regularly?**				
Always	74	17.5	3	1 (2–3)
Never	2	0.5		
Rarely	135	32.0		
Sometimes	211	50.0		
**Do you keep a record of the patient's blood pressure readings?**				
Always	295	69.9	4	1 (3–4)
Never	3	0.7		
Rarely	32	7.6		
Sometimes	92	21.8		
**Do you communicate with healthcare professionals about the patient's hypertension management?**				
Always	254	60.2	4	1 (3–4)
Never	1	0.2		
Rarely	40	9.5		
Sometimes	127	30.1		
**How often do you accompany the patient to doctor's appointments?**				
Always	79	18.7	3	0 (3–3)
Never	4	0.9		
Rarely	74	17.5		
Sometimes	265	62.8		
**How often do you prepare meals for the patient that meet dietary guidelines for hypertension?**				
Always	70	16.6	3	1 (2–3)
Never	8	1.9		
Rarely	157	37.2		
Sometimes	187	44.3		
**Do you help the patient with meal planning?**				
Always	65	15.4	2	1 (2–3)
Never	25	5.9		
Rarely	214	50.7		
Sometimes	118	28.0		

Note: IQR: Interquartile range.

### 3.5 Correlation between KAP regarding hypertension

To examine the link between the KAP scores, a bivariate analytic model was used to determine the correlation. Knowledge exhibited a substantial fair, positive connection with attitude (r = +0.255; p < 0.001) and practice (r = +0.252; p < 0.001). While there was no correlation between attitude and practice (r = +0.064; p = 0.190) (Table 5).

**Table 5:** Pearson Correlation Test, correlations between knowledge, attitude, and practice about hypertension (n = 422).

**Variables**	***r*-value**	***p*-value**	**Interpretation**
**Knowledge and Attitude**	+0.255	<0.001	Fair, positive correlation
**Knowledge and Practice**	+0.252	<0.001	Fair, positive correlation
**Attitude and Practice**	+0.064	0.190	No correlation

### 3.6 Correlation of KAP scores with age and duration in care regarding hypertension

**Fig. 1:**
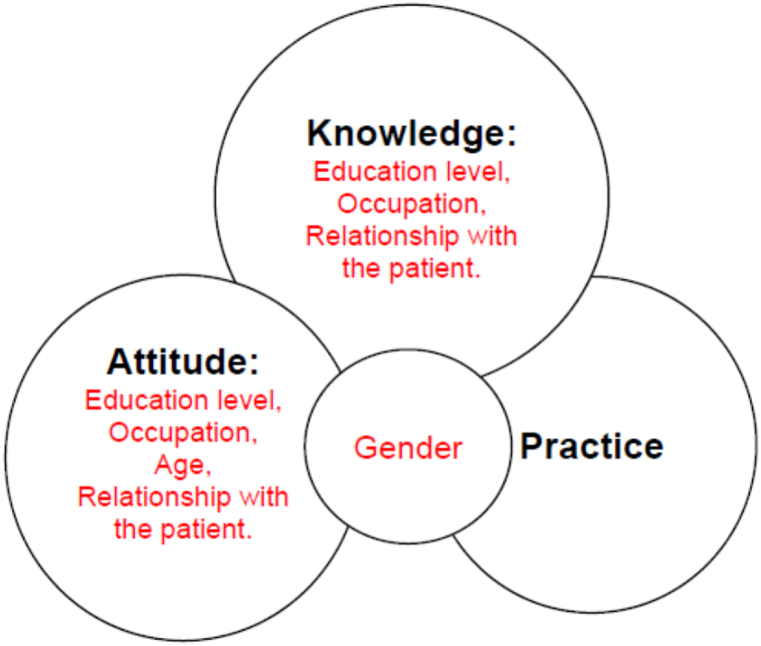
Determinants of KAP among caregivers of hypertensive patients.

To assess the relationship between age and the KAP scores associated with hypertension, a bivariate analysis model was utilized to quantify the correlation. Age did not correlate with either the knowledge score (r = +0.034; p = 0.490) or the practice score (r = +0.043; p = 0.382). While age and attitude score showed a substantial fair, positive correlation (r = +0.233; p < 0.001). Similarly, to investigate the association between the length of stay in care and the KAP scores for hypertension, the correlation was evaluated using a bivariate analysis model. There was no link found between the duration in care and the knowledge, attitude, and practice scores (r = +0.024; p = 0.627), (r = −0.089; p = 0.066), and (r = +0.083; p = 0.089), respectively (Table 6, Fig. 1).

**Table 6:** Bivariate analysis of correlations of KAP with age and duration in care (n = 422).

**Variables**	**Age**			**Duration in Care**		
	***r*-value**	***p*-value**	**Interpretation**	***r*-value**	***p*-value**	**Interpretation**
**Knowledge**	+0.034	0.490	No correlation	+0.024	0.627	No correlation
**Attitude**	+0.233	<0.001	Fair, positive correlation	−0.089	0.066	No correlation
**Practice**	+0.043	0.382	No correlation	+0.083	0.089	No correlation

### 3.7 Effect of gender, educational level, occupation, and caregiver- patient relationship on KAP

Using Mann-Whitney *U* test, we analyzed the effect of demographic characteristics on KAP scores. The demographic characteristics included gender, males (n = 155) and females (n = 267); occupation, non-employed participants (n = 264) included farmers, students, and housewives, employed participants (n = 158) included businesspeople and those employed in various sectors; education level, with higher levels of education (n = 135) including secondary and college education, and lower levels of education (n = 287) including those with no education and only primary education; as well as relationship with the patient, participants with blood relationship (n = 153) included the patient's parent and child, while those without blood relationship (n = 269) included spouses and other individuals. All the four groups showed a notable effect on knowledge and attitudes regarding hypertension, with p-values ranging from <0.001 to 0.034, and *r* ranging from 0.107 to 0.453. With a p-value of 0.016 and *r* 0.118, gender showed an effect on practice, nevertheless, with p-values of more than 0.05, ranging from 0.122 to 0.803 and *r* below 0.1, indicated that the other social demographic characteristics under investigation had no effect on practice (Table 7, Fig. 1)

**Table 7:** Effect of social demographic characteristics on knowledge, attitude and practices regarding hypertension Mann-Whitney *U* test (n = 422).

**Knowledge**							
**Groups being compared**		**n**	**Median**	**U**	**Z**	***r***	***p-*value**
**Gender**	Male	155	10	17203.500	−2.941	0.143	0.003^a^
	female	267	9				
**Education** **Level**	Lower	287	9	12634.500	−5.871	0.286	<0.001^a^
	Higher	135	10				
**Occupation**	Non employed	264	9	15467.500	−4.525	0.220	<0.001^a^
	Employed	158	10				
**Relationship**	Blood relation	153	9	18264.0000	−2.116	0.103	0.034^a^
	Non-blood relation	269	9				
**Attitude**							
**Groups being compared**		**n**	**Median**	**U**	**Z**	***r***	***p-*value**

**Gender**	Male	155	18	15479.0000	−4.594	0.224	<0.001^a^
	female	267	16				
**Education** **Level**	Lower	287	16	13134.500	−5.680	0.276	<0.001^a^
	Higher	135	18				
**Occupation**	Non employed	264	16	10258.0000	−9.301	0.453	<0.001^a^
	Employed	158	18				
**Relationship**	Blood relation	153	16	18087.0000	−2.201	0.107	0.028^a^
	Non-blood relation	269	17				
**Practice**							
**Groups being compared**		**n**	**Median**	**U**	**Z**	***r***	***p-*value**

**Gender**	Male	155	25	17800.0000	−2.414	0.118	0.016^a^
	female	267	25				
**Education** **Level**	Lower	287	25	19083.0000	−0.250	0.0122	0.803
	Higher	135	25				
**Occupation**	Non employed	264	25	19940.0000	−0.762	0.037	0.446
	Employed	158	25				
**Relationship**	Blood relation	153	25	18729.500	−1.548	0.075	0.122
	Non-blood relation	269	25				

Note.

a Statistically significant at p < 0.05, 2 tailed.

## 4 Discussion

This cross-sectional study aimed to assess knowledge, attitude, and practices towards hypertension among caregivers of hypertensive patients and to identify factors that influence the KAP, found that 84.8 % of the caregivers had little knowledge regarding hypertension, and almost all the caregivers (97.8 %) got their knowledge from the healthcare provider, which is different from the study conducted in Bangladesh, where most of the caregivers got the information from the internet and other platforms [8], indicating the state of poverty and illiterate in which the study population is and the importance of technology in advancing health care service delivery and improving disease outcomes. This also shows that health care providers don't have enough time to give formal health education to the caregivers on diseases due to work overload [27]. Implementing structured educational programs, fostering community awareness and engagement, and conducting regular follow-ups can significantly bridge the knowledge gap. The study showed that most caregivers were spouses (63.0 %), followed by children (25.8 %) of the patients. This is also the case in studies conducted in Bangladesh and Nigeria about caregivers of hypertensive patients [5,8], as these are the family members that are always close by. More than half (61.4 %) of the patients being taken care of have been in care for at least 1–5 years. Despite this lengthy duration, the caregivers' knowledge remains inadequate. This is surprising, as one would typically expect that a longer caregiving period would lead to improved knowledge and understanding [8].

### 4.1 KAP regarding hypertension

Participants demonstrated poor knowledge about hypertension, achieving an average score of only 38 %. Alarmingly, just 10.2 % of participants could accurately identify the risk factors associated with hypertension, and a mere 3.1 % recognized its potential complications. Comparative research from Lebanon reported a knowledge score of 47.3 %, while Saudi Arabia's score was slightly higher at 47.1 %. Studies conducted in Ghana and various regions across East and West Africa also revealed knowledge scores below 40 %, similarly revealed low levels of understanding among caregivers and communities, highlighting that inadequate knowledge of hypertension is a widespread issue across different demographics [10,13,19,28]. In contrast, a study in Bangladesh (54.83 %) and Jordan (70 %) found that caregivers of hypertensive patients had a significantly better knowledge [7,8]. This discrepancy is attributed to the fact that most respondents in the Bangladeshi and Jordan studies were from urban areas, where access to information is more readily available and literacy rates are higher. In contrast, our study focused on rural populations, where access to information is limited and illiteracy rates are higher [13]. This situation highlights the urgent need for interventions aimed at improving access to information and addressing the knowledge gap through targeted health education initiatives.

According to the participants’ responses, excessive salt intake was the most mentioned risk factor, and some attributed the disease to stress. These results are in line with those studies conducted in Benin, Philippines, China, Zimbabwe, and Bangladesh [23,[29], [30], [31], [32]], in which most of the participants indicated excessive salt intake and stress as risk factors. Few participants (15.4 %) correctly answered questions about the preventive measures of hypertension. This is consistent with research done in Saudi Arabia which found low levels of knowledge on prevention of stroke which is a complication of hypertension [33]. Unlike in Bangladesh study where caregivers were able to know the normal blood pressure and were able to monitor it [8], almost all the participants (95.6 %) did not know how often blood pressure should be monitored, while 46.2 % knew the normal blood pressure measurement. This indicates that patients risk developing complications due to limited knowledge regarding blood pressure monitoring among caregivers [12]. Half of the participants (51.9 %) were able to mention signs and symptoms of hypertension like in the Bangladesh study. This emphasizes the significance of knowledge in identifying high blood pressure early as one of the key elements in reducing the rate at which hypertension develops [34].

Participants displayed an excellent attitude towards hypertension, achieving an average score of 93.3 %. Notably, 98.3 % recognized hypertension as a serious condition, and 95 % believed it could be managed through medication and lifestyle changes. This finding contrasts sharply with a study conducted in Bangladesh, which reported an attitude score of only 47.95 % among caregivers [8]. While the participants in this study demonstrated limited knowledge about hypertension, they nonetheless acknowledged the importance of effectively managing the condition.

This gives a window of opportunity to enhance hypertension prevention and management strategies in the communities regardless of their poor knowledge. These findings are consistent with a scoping review of hypertension in Sub-Saharan Africa which found out that most of the communities have good attitudes regarding hypertension [35]. Almost half (46.4 %) did not feel having enough information about hypertension, while (44.8 %) were not confident in caring for hypertensive patients, hence the need for their involvement in all activities in the management of hypertensive patients. Nonetheless, 96.9 % felt supported by the health care professionals in their caring role. This is the outcome of community health workers' participation in Malawi's hypertension management program [36].

Participants demonstrated good practices related to hypertension prevention and management, achieving a mean score of 78.7 %. Specifically, 74.6 % of participants ensured medication adherence, while varying percentages engaged in lifestyle modifications and monitoring practices. This finding aligns with research conducted in Bangladesh, which reported a practice score of 61.26 % [8]. This comparison highlights an important trend that despite gaps in knowledge, individuals often exhibit good practices in preventing and managing hypertension. These results emphasize the necessity for targeted educational interventions aimed at enhancing knowledge while sustaining excellent attitude and good practices in hypertension prevention and management across diverse populations.

Medication adherence was good at 74.6 % similar to another previous study conducted in Malawi [37], which denotes the severity of the disease as one of the factors leading to good medication adherence. Only a few participants (17.5 %) could monitor patients' blood pressure regularly which is in line with research conducted in Saudi Arabia [33], where communities had poor blood pressure monitoring. This could be due to the study setting and health systems across LMICs, where health facilities are far from reach [38]. This puts communities at risk as there are a lot of gains when hypertension is diagnosed early. Only a few (18.7 %) were able to accompany the patient to doctor's appointments, this is because most of the patients were stable and were back to their day-to-day activities. Less than a quarter (16.6 %) and (15.4 %) of participants were able to prepare meals that met dietary guidelines and managed to help patients with meal planning respectively. This is because of poverty, low socioeconomic status, and food insecurity faced by the participants, resulting in eating whatever is available [39]. There is a need to incorporate food security in the prevention and management of NCDs in LMICs as this may improve availability of health diet within the communities.

### 4.2 Correlation between KAP regarding hypertension

The cumulative knowledge and attitude scores, as well as the cumulative knowledge and practice scores pertaining to hypertension, showed a fair positive connection. It was shown that higher knowledge was linked to a more positive attitude about hypertension among the participants. This outcome suggests that individuals possessing a greater understanding of hypertension tend to exhibit a more positive attitude towards hypertension prevention and management. The observed findings align with a study in Malaysia that identified a noteworthy correlation between knowledge and attitude related to hypertension within the studied population [40]. The study also found a strong positive correlation between knowledge and the implementation of practices related to hypertension among the respondents. A clear link exists between possessing accurate knowledge and adopting appropriate practices. For instance, individuals' awareness that excessive salt intake is linked to elevated blood pressure tends to reduce their salt consumption [10]. Conversely, individuals who wrongly thought that regular alcohol use did not increase the risk of hypertension were more inclined to overindulge in alcohol [19]. It's interesting to note that within the study population, there was no significant association found between attitude and practice regarding hypertension which is contrary to research conducted in Lebanon and Ethiopia which found a fair positive correlation between attitude and practice [19,41]. This implies that better attitudes could result in something different than good practices. For example, even though most responders agreed that a healthy diet and increased physical activity could help manage hypertension, they did not aggressively encourage their patients to adopt these lifestyle changes. This could be due to poor knowledge and the low social economic status which prohibits people from adopting lifestyle changes such as eating healthy diets and most of these people are farmers and farming is a physical activity on its own [24].

### 4.3 Determinants of knowledge, attitude, and practice

While research done in Bangladesh and Lebanon found negative correlation between age and knowledge and no correlation between age and attitude; and age and practice, our study findings show that age correlated with attitude regarding hypertension in the population under study [10,19]. Interestingly, no such correlation was observed concerning hypertension-related knowledge and practices scores. This implies that as individuals age, their attitude toward hypertension tends to become more favorable, yet their knowledge and actual practices in managing hypertension remain unaffected. This highlights the need to consider age in the planning and execution of health care strategies because as people grow old, they tend to forget easily hence a need for continued health education and their weak body may inhibit them from doing physical activities. Notably, in this study, Knowledge, Attitude, and Practice (KAP) levels were unaffected by the length of time the patient received care. Despite the varying durations in care, there was no significant difference in the KAP scores among the respondents. This may be because the respondents were caregivers and not patients themselves [19].

Furthermore, the study unveiled a noteworthy gender effect on KAP concerning hypertension in the study population demonstrating the role women take in caring for the sick in most of the LMICs. In contrast, factors such as educational level, occupation, and the relationship with the patient demonstrated an effect on knowledge and attitude, but no effect on practices related to hypertension. These results are similar to those found in studies conducted in Bangladesh, Lebanon, Saudi Arabia [10,19,33]. The fact that education level affected the KAP was expected as higher education increases awareness regarding health issues and provides access to the required information [42]. This highlights the relationship of different factors which help in shaping individuals' perceptions and behaviors toward hypertension, emphasizing the need for a clear approach in addressing these variety of aspects [43]. The closer the relationship and the higher the occupation the better the understanding and perception of diseases.

Studies that have been conducted in Malawi shows that hypertension prevalence increases with age in both genders [44]. Managing lifestyle choices is crucial for the prevention of hypertension, as combining these factors with age escalates the risk of cardiovascular disease [45]. Therefore, directing efforts towards organizing educational programs that address life style changes, with a primary emphasis on reaching caregivers, older populations, and rural communities, can contribute significantly in elevating the Knowledge, Attitude, and Practices (KAP) levels [46]. Additionally, it is crucial to tailor awareness, treatment, and prevention strategies to align with the specific needs of each community and country [13].

### 4.4 Limitations of the study

The study utilized a systematic random sampling and the study area which is a rural setting raising concerns about the generalizability of the findings to the broader population and also, self-reporting of the participants may have resulted in social desirability bias in which respondents conceal their true opinion on a subject in order to make themselves look good to other. Nevertheless, these results can be applied in a rural setting with limited resources like Neno, Malawi. Additionally, the difference in the duration of care of the patients may have introduced recall bias. Future research should explore both urban and rural areas as well as simple random sampling techniques for the results to be generalizable.

## 5 Conclusions

This study revealed excellent attitude and good practices among caregivers yet highlighted concerns arising from their poor knowledge scores. It is imperative to address this crucial issue. Our data highlights the necessity for targeted interventions, particularly among caregivers who are less educated, unemployed, older, and influenced by gender factors, as these variables determine their Knowledge, Attitude, and Practice (KAP) scores. Consequently, our findings advocate for implementing tailored learning programs designed for caregivers and communities, aimed at bridging knowledge gaps, enhancing awareness, and preventing emergencies through improved practices. Furthermore, there is a compelling need for comprehensive health education initiatives within the communities to foster lifestyle changes. This proactive approach is essential to mitigate potential complications associated with the disease, emphasizing the importance of preventing adverse outcomes through increased community understanding.

## CRediT authorship contribution statement

**Chikondi Maluwa:** Writing – original draft, Visualization, Software, Resources, Methodology, Investigation, Funding acquisition, Data curation, Conceptualization. **Sitalire Kapira:** Writing – review & editing, Software, Investigation, Data curation. **Hataichanok Chuljerm:** Writing – review & editing, Resources, Investigation, Conceptualization. **Wason Parklak:** Writing – review & editing, Resources, Investigation. **Kanokwan Kulprachakarn:** Writing – review & editing, Supervision, Resources, Funding acquisition, Conceptualization.

## Data availability statement

The data presented in this study are available from all authors.

## Funding

This work was (partially) supported by Research Institute for Health Sciences, 10.13039/501100002842Chiang Mai University, grant number 003/2566 and 10.13039/501100020857Thailand International Cooperation Agency (10.13039/501100020857TICA), Thailand International Postgraduate Program (TIPP), grant number Kor Kor: 1602.1/4318. This research project was supported by Fundamental Fund 2024, 10.13039/501100002842Chiang Mai University. The study's design, data collection, analysis, interpretation, and paper writing were all done independently of the sponsoring organizations.

## Declaration of competing interest

The authors declare the following financial interests/personal relationships which may be considered as potential competing interests:Kanokwan Kulprachakarn reports financial support was provided by 10.13039/501100002842Chiang Mai University. Chikondi Maluwa reports financial support was provided by 10.13039/501100020857Thailand International Cooperation Agency (10.13039/501100020857TICA). If there are other authors, they declare that they have no known competing financial interests or personal relationships that could have appeared to influence the work reported in this paper.
